# Influence of Acidic pH on Wound Healing In Vivo: A Novel Perspective for Wound Treatment

**DOI:** 10.3390/ijms232113655

**Published:** 2022-11-07

**Authors:** Pivian Sim, Xanthe L. Strudwick, YunMei Song, Allison J. Cowin, Sanjay Garg

**Affiliations:** 1 Centre for Pharmaceutical Innovation (CPI), Clinical and Health Sciences, University of South Australia, Adelaide, SA 5000, Australia; 2 Regenerative Medicine, Future Industries Institute, University of South Australia, Adelaide, SA 5095, Australia

**Keywords:** acidic, citric acid, phosphoric acid, pH, healing, wound, closure, re-epithelialization, collagen, excisional

## Abstract

There has been little understanding of acidification functionality in wound healing, highlighting the need to study the efficacy of wound acidification on wound closure and cellular activity in non-infected wounds. This study is focused on establishing the healing potential of wound acidification in non-infected wounds. Acidic buffers, constituting either phosphoric or citric acid, were employed to modify the physiological pH of non-infected full-thickness excisional murine wounds. Acidification of the wound by acidic buffers was found to be an effective strategy to improve wound healing. A significant improvement in wound healing parameters was observed as early as 2 days post-treatment with acidic buffers compared to controls, with faster rate of epithelialization, wound closure and higher levels of collagen at day 7. pH is shown to play a role in mediating the rate of wound healing, with acidic buffers formulated at pH 4 observed to stimulate faster recovery of wounded tissues than pH 6 buffers. Our study shows the importance of maintaining an acidic wound microenvironment at pH 4, which could be a potential therapeutic strategy for wound management.

## 1. Introduction

Wound healing is a complex process that can be categorized into four integrated and overlapping phases involving hemostasis, inflammation, proliferation and connective tissue remodeling as an innate response to a trauma to regenerate a functional epidermal barrier [1,2]. There are many local wound factors and systemic mediators that are known to influence this healing process [3]. The wound pH milieu is one important local factor that is often overlooked, which has been commonly described as a function for the skin barrier. The importance of regulating the pH of the wound milieu to improve wound healing is emerging. We have begun to understand its importance in mediating many biochemical processes that stimulate tissue healing properties [4,5,6,7].

The natural physiological pH of a healthy human epidermal layer is maintained at a slightly acidic milieu with the surface of the skin in the range of 4.5–5.3 and increasing pH gradient over the horny layer to 6.8 as it reaches the lower stratum corneum [8,9]. The microenvironment pH of wounds is naturally more alkaline as the presence of a trauma disturbs this acidic milieu and exposes underlying tissues that have a physiological pH of 7.4 [4,10,11]. The microenvironment pH of wounds can vary depending on the type of wound, for example, acute wounds are reported to have a mean pH of 7.44 and chronic wounds at a range from pH of 7.42 to 8.90 [4,12]. Such alkaline microenvironments are favorable for bacterial growth, thus posing as the primary strategy for many wound management regimens using acids to prevent microbial growth [13,14].

As the wound heals, physiological mechanisms begin to naturally restore an acidic milieu, progressing from a neutral pH to more acidic microenvironment throughout the healing process [10,15]. The restoration of this acidic milieu acts as a natural physiological response to mediate various internal cellular processes aimed to restore the epidermal barrier that helps facilitate wound oxygenation levels, influencing macrophage and fibroblast activity and enzymatic activity participating in wound healing [16,17]. Oxygen tension within the wound is a systemic factor that has been reported to be strongly influenced by wound pH [18,19,20,21], whereby hemoglobin releases more oxygen under acidic microenvironment due to the Bohr effect [22]. As oxygen is an essential component for the growth of fibroblast cells and collagen synthesis during wound healing [19], fibroblast cells have been reported to be more active in an acidic microenvironment expressing faster migration and proliferation behavior [23,24]. Increased activity of fibroblast cells promote rapid epithelialization, wound contraction and angiogenesis, leading to faster wound regeneration [25,26]. Such increases in fibroblast proliferation and migration behaviors under acidic conditions are thought to be strongly associated to polarity and epithelial potential (EP) between wounded and unwounded tissues, which are strongly influenced by microenvironmental pH. The fluctuation of epithelial potentials generated during wounding helps guide these cells to each other, leading to faster wound closure [27,28].

Many studies assessing wound acidification have focused primarily on modifying the physiological pH of wounds for microbial management in chronic wounds indications [29,30,31,32]. However, there has been little understanding of how acidification functionality affects wound closure and cellular activity, highlighting the need to firstly study the effect of acids in non-infected wound models to determine their efficacy in wound healing. The present study employed the use of acidic buffers to modify the physiological pH of non-infected, full-thickness, excisional murine wounds. Acidic buffers were formulated with the main constituent being either phosphoric or citric acid at different pH of 4 and 6. These acids were chosen for phosphoric acid’s natural occurrence in the body and its physiological role in regulating cell division, growth and development [33,34], and citric acid, due to its physiological role in the tricarboxylic acid cycle (TCA) regulating cell proliferation and metabolism [35,36]. This study aims to demonstrate the in vivo efficacy of acidification treatment following a treatment regimen and investigate its effect on the re-epithelialization rate and collagen deposition in treated wound tissues.

## 2. Results

### 2.1. Acidic Buffers Increased Rate of Wound Closure in Murine Skin

**Figure 1:**
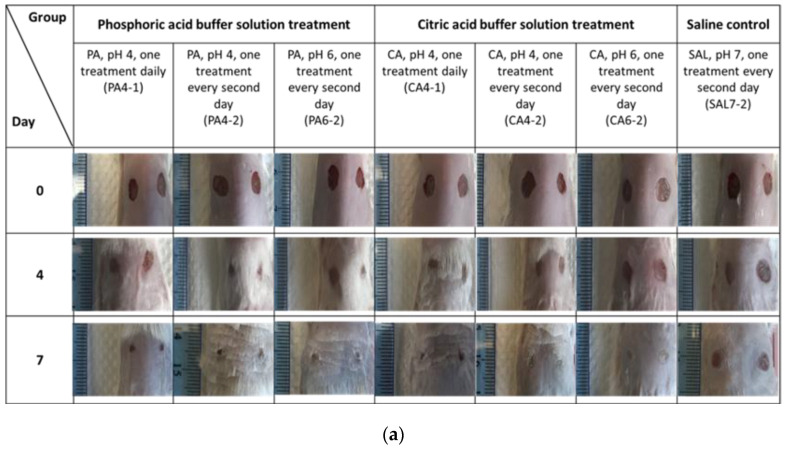

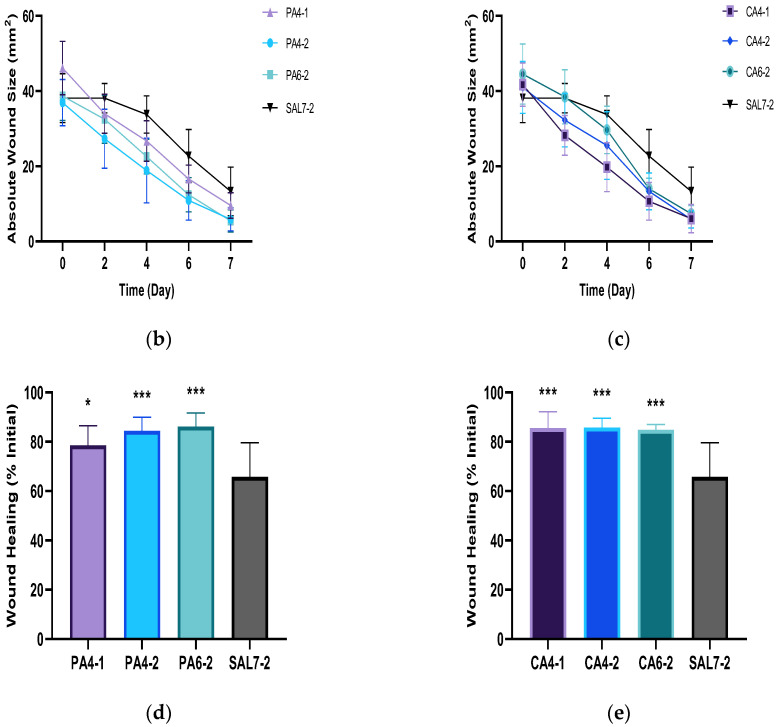
(**a**) Macroscopic evaluation in relation to the time course of gross wound healing after one week of topical application of phosphoric acid (PA) and citric acid (CA) buffer solutions. (**b**) Differences in absolute wound area between phosphoric acid pH 4, once-daily treatment (PA4-1), phosphoric acid pH 4 and 6, once-every-second-day treatment (PA4-2 and PA6-2), and saline control group (SAL7-2). (**c**) Time course of wound regeneration for animal model after dermal treatment with citric acid pH 4, once-daily treatment (CA4-1), citric acid pH 4 and 6, once-every-second-day treatment (CA4-2 and CA6-2) compared with saline control group (SAL7-2). (**d**) Macroscopic wound healing on day 7 when animals were topically applied with phosphoric acid with pH values of 4 and 6, and with different dosing regimens. (**e**) Topical administration of citric acid buffer solutions in comparison to saline as control group at day 7 post-treatment. Each bar represents mean ± standard deviation. * *p* < 0.05 and *** *p* < 0.001 indicates the results obtained were statistically significant from control group (SAL7-2).

The rate of wound closure following acidic treatment with low ionic strength (0.01 M) phosphoric acid and citric acid buffers at pH of 4 and 6 were assessed using macroscopic analysis of treated full-thickness excisional wounds on mice. Wounds treated topically with either phosphoric acid or citric acid buffer solutions were observed to have a significantly higher rate of wound closure, with *p*-value less than 0.0001 for all treatment groups in comparison to control solution (saline) by 7 days post-wounding (Figure 1a–c). Macroscopic evaluation of wounds, following treatment every second day with phosphoric acid pH 4 and pH 6 (PA4-2 and PA6-2), resulted in significantly smaller absolute wound size (mm^2^) of 5.89 ± 3.07 and 5.48 ± 2.93, respectively, in comparison to a wound size of 13.34 ± 6.46 mm^2^ from the saline control group (SAL7-2). Wound pH of saline control groups was measured at 7.52 ± 0.24 on day 0, 7.46 ± 0.27 on day 2, 7.20 ± 1.87 on day 4, 7.14 ± 0.31 on day 6 and 7.11 ± 0.28 by day 7. Increasing treatment regime to daily application (PA4-1) did not result in further reduction of wound size, with wounds maintaining at approximately 9.60 ± 3.43 mm^2^. However, treatment utilizing phosphoric acid buffers, irrespective of dosing frequency, had significantly decreased the wound size in the animal model at day 7 post-treatment, with *p*-value less than 0.0001.

The absolute wound size (mm^2^) of the animal model treated with citric acid at pH 4 once-every-second-day (CA4-2) had successfully reduced to 5.87 ± 2.29. Increasing the dosing frequency of citric acid pH 4 to once-daily (CA4-1) did not further reduce the wound size (6.09 ± 3.78). Wounds treated with pH 6 every second day (CA6-2) were slightly larger, measured at 7.37 ± 2.17. Nevertheless, citric acid buffer-treated wounds were still significantly smaller than the saline control group (SAL7-2). Statistically significant results were obtained from two-way ANOVA for PA4-2 (*p*-value = 0.0004), PA6-2 (*p*-value = 0.0344), CA4-1 (*p*-value < 0.0001) and CA4-2 (*p*-value = 0.0419) on day 2. Apart from CA6-2, all other treatment groups were statistically significant on day 4, with *p*-value < 0.0001 for PA4-2, PA6-2 and CA4-1, followed by PA4-1 (*p*-value = 0.0079) and CA4-2 (*p*-value = 0.0213). On day 6, PA4-2 and CA4-1 were the most statistically significant, with *p*-values = 0.0003 and 0.0002, respectively, followed by PA6-2 (*p*-value = 0.0010, CA4-2 (*p*-value = 0.0028) and CA6-2 (*p*-value = 0.00390). These results showed that buffered treatment groups improved wound healing as early as day 2 post-treatment.

Mean percentages of wound healing were calculated from initial wound size measurements at day 0 compared to the size of collected wound tissue after 7 days post-treatment, using the macroscopic analysis method. Results showed significantly better percentage healing in acid-treated wounds compared to the saline control group, as shown in Figure 1d,e. The mean percentage wound healing (% of initial) of phosphoric acid groups for PA4-1, PA4-2, PA6-2 over 7 days of treatment were measured at 78.54 ± 7.71, 84.36 ± 6.02, 86.09 ± 5.68, respectively. For citric acid, mean percentages of wound healing (% of initial) of treated groups for CA4-1, CA4-2, CA4-6 were measured at 85.53 ± 3.59, 85.74 ± 2.61, 84.79 ± 2.36, respectively. In comparison, the mean percentage wound healing for SAL7-2 control group was slower, measuring at 65.02 ± 13.83 on day 7. The *p*-values of measured mean percentage of wound healing for all phosphoric acid (apart from PA4-1 with *p*-value = 0.0265) and citric acid treatment groups were less than <0.0001.

### 2.2. Acidic Microenvironment Improves Rate of Re-Epithelialization and Wound Contraction

**Figure 2:**
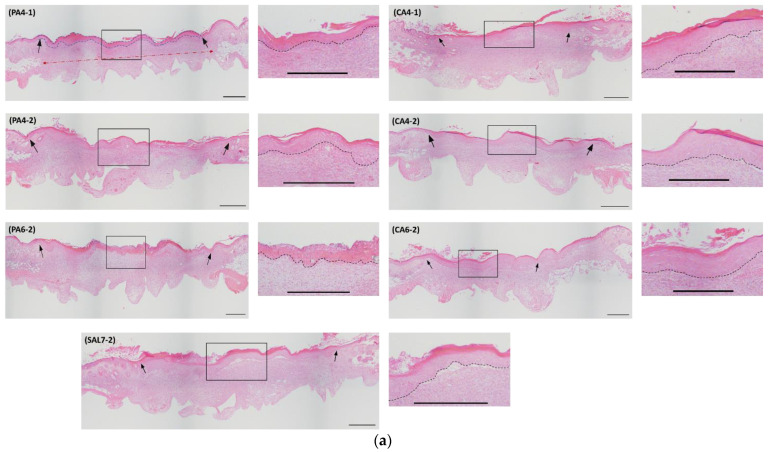

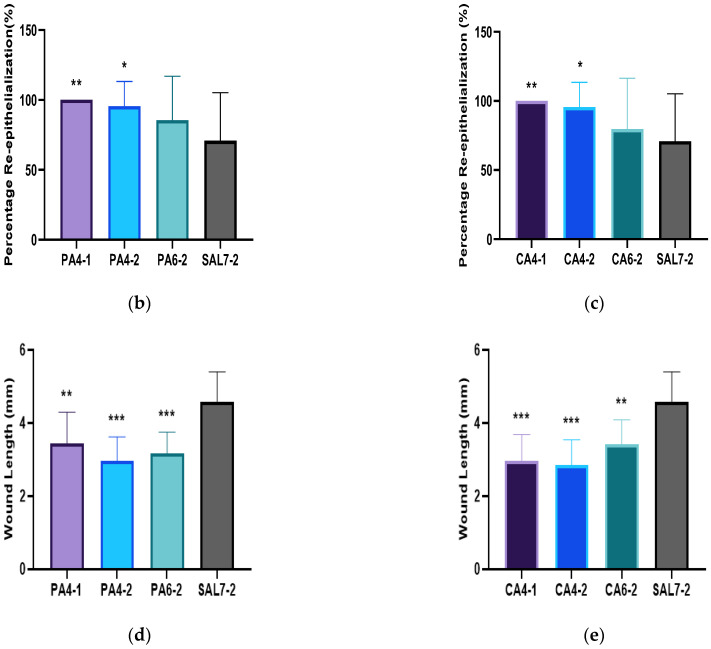
Microscopic analysis confirmed by hematoxylin and eosin (H&E) staining of percentage wound recovery after one week of topical administration of phosphoric acid (PA) and citric acid (CA) buffered solutions with different pH values and treatment regimes. (**a**) Full-thickness punch biopsy sample obtained from animal model in all acidic buffered treatment groups and saline control group at Day 7 post-injury. Original magnification 4×. Black scale bar represents 500 µm. Black arrows indicate the width of new tissue formation, blue dotted line indicate the re-epithelialized area, red dotted line indicate the panniculus gap measurement, and black dotted line indicate the base of epithelium. (**b**) Percentage re-epithelialization of animal model following topical treatment with phosphoric acid buffer solutions of pH 4 and pH 6. (**c**) Topical administration of citric acid buffer solutions or saline as control group. (**d**) Measurement of wound length at Day 7 post-injury following topical treatment with phosphoric acid buffer solutions of pH 4 and pH 6. (**e**) Wound length obtained from animal model after topically treated with citric acid buffer solutions with pH adjusted to 4 and 6 for 7 days. Each bar represents mean ± standard deviation. * *p* < 0.05, ** *p* < 0.01 and *** *p* < 0.001 indicates the results obtained were statistically significant from control group (SAL7-2).

Histological analysis of collected wound tissue samples was performed to determine the percentage of re-epithelialization, epithelial thickness and width of panniculus gap of wound tissues collected 7 days post-wounding, stained using hematoxylin and eosin (H&E). Histological evaluation of wound re-epithelialization showed wounds treated with pH 4 phosphoric and citric acid possessed faster re-epithelialization compared to pH 6 buffers and control saline solution, as shown in Figure 2. Percentage re-epithelialization (%) of wound tissues treated with pH 4 acidic buffers following a once-daily treatment regimen (PA4-1 and CA4-1) reached 100% re-epithelialization, with *p*-value of 0.0046 and 0.0084, respectively (Figure 2b,c). In contrast, wounds that were treated following a once-every-second-day treatment regimen (PA4-2 and CA4-2) and pH 6 acidic buffers (PA6-2 and CA6-2) showed slower wound re-epithelialization. Percentage re-epithelialization of these treatment groups for PA4-2 was measured at 95.61 ± 17.58 (*p*-value = 0.0195) and PA6-2 was measured at 85.62 ± 31.37 (*p*-value > 0.005) from tissues collected 7 days post-operatively. For citric acid treatments, percentage re-epithelialization of wound tissues following every second day treatment regimen (CA4-2) was measured at 95.51 ± 17.97 (*p*-value = 0.0303) and pH 6 (CA6-2) was measured at 79.70 ± 34.66 (*p*-value > 0.05). The saline control group (SAL7-2) showed the slowest percentage of re-epithelialization, measured at 70.83 ± 34.36. The percentage of wounds that were fully re-epithelialized was higher in treatment groups compared to saline control, at 100% for PA4-1, 94% for PA4-2, 81% for PA6-2, 100% for CA4-1, 94% for CA4-2 and 85% for CA6-2, compared to 67% for SAL7-2 7 days post-operatively.

Wound length of tissue samples was also measured at 7 days post-wounding, as an indication of wound healing influenced by re-epithelialization and contraction. Results showed significantly smaller wound length in acid-treated wounds compared to the saline control group, as shown in Figure 2d,e. The mean wound lengths (mm) of phosphoric acid groups for PA4-1, PA4-2 and PA6-2 were measured at 3.44 ± 0.84, 2.98 ± 0.63 and 3.17 ± 0.57, respectively. For citric acid, mean wound lengths (mm) of treated groups for CA4-1, CA4-2 and CA6-2 were 2.96 ± 0.70, 2.85 ± 0.68 and 3.42 ± 0.65, respectively, when compared to wound length of saline-treated group of 4.60 ± 0.79. All acidic buffer solution treatment groups were found to be statistically significant in comparison to saline control group, with *p*-values less than 0.01.

**Figure 3:**
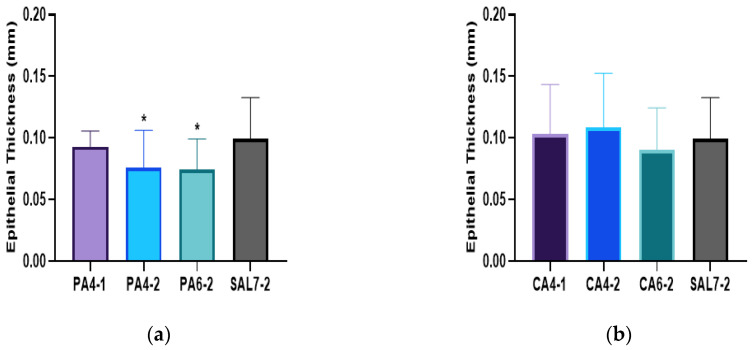
Comparison of epithelial thickness as an indication of the quality of regenerated wound between (**a**) phosphoric acid (PA) buffered solution treatment groups on day 7 and (**b**) citric acid (CA) buffered solution treatment groups on day 7 post-wounding with saline control group (SAL7-2). Each bar represents mean ± standard deviation. * *p* < 0.05 indicates the results obtained were statistically significant from control group (SAL7-2). PA4-1 n = 14/14, PA4-2 n = 15/16, PA6-2 n = 13/16, CA4-1 n = 16/16, CA4-2 n = 15/16, CA6-2 n = 12/14 and SAL7-2 n = 8/12.

The epithelial thickness of fully re-epithelialized wound tissues was also assessed as a defining parameter to wound recovery. Epithelial thickness of all wound tissues treated with acidic buffers displayed better recovery, showing almost complete regeneration of the epithelial layer evidence by surrounding wounded tissues, as shown in Figure 3. The epithelial thickness of treated wound tissues by phosphoric acid (PA) pH 4 treatment showed similar epithelial thickness by day 7, measuring at 0.09 ± 0.01 mm for PA4-1, 0.08 ± 0.03 mm for PA4-2 and 0.07 ± 0.02 mm for PA6-2. Both citric acid (CA) pH 4 treatments (CA4-1 and CA4-2) were measured at 0.10 ± 0.04 mm, and citric acid pH 6 (CA6-2) showed a thickness of 0.09 ± 0.03 mm. The control saline group (SAL7-2) showed the thickest epithelial layer at day 7, measuring at 0.11 ± 0.03 mm. Significantly thinner epithelial thickness was observed following treatment of PA4-2 and PA6-2, with *p*-values of 0.0384 and 0.0354, respectively.

**Figure 4:**
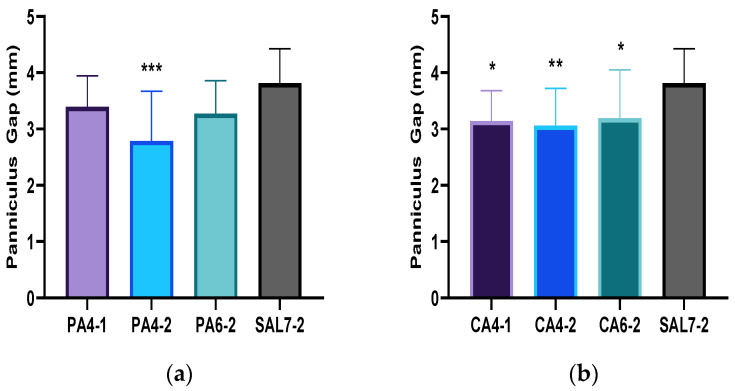
Panniculus carnosus muscle gap measurement during wound healing process when treated with (**a**) phosphoric acid (PA) buffered solutions of pH 4 and pH 6, (**b**) citric acid (CA) buffered solutions of pH 4 and pH 6 in comparison to saline control group (SAL7-2). Each bar represents mean ± standard deviation. * *p* < 0.05, ** *p* < 0.01 and *** *p* < 0.001 indicates the results obtained were statistically significant from control group (SAL7-2).

Histological analysis of wound-contraction by measurement of the gap between the striated panniculus carnosus muscle was also assessed to understand the effect of acidic buffer solutions on wound contraction. The rate of wound contraction was significantly faster for PA4-2 (*p*-value = 0.0003), CA4-1 (*p*-value = 0.0201), CA4-2 (*p*-value = 0.0079) and CA6-2 (*p*-value = 0.0419) in comparison to control saline group. Panniculus gap measurement of phosphoric acid (PA) treated wound tissues was measured at 3.39 ± 0.55 mm (PA4-1), 2.79 ± 0.88 mm (PA4-2) and 3.27 ± 0.58 mm (PA6-2), respectively. Citric acid (CA) treatment groups were measured at 3.14 ± 0.54 mm for CA4-1, 3.06 ± 0.66 mm for CA4-2 and 3.19 ± 0.86 mm for CA6-2. Panniculus gap measurement of control saline group (SAL7-2) treatment showed the widest gap in comparison to all other acidic treatment groups measured at 3.81 ± 0.61 mm (Figure 4).

### 2.3. Acidic Treatments Promote Collagen Deposition in Wounded Tissues

**Figure 5:**
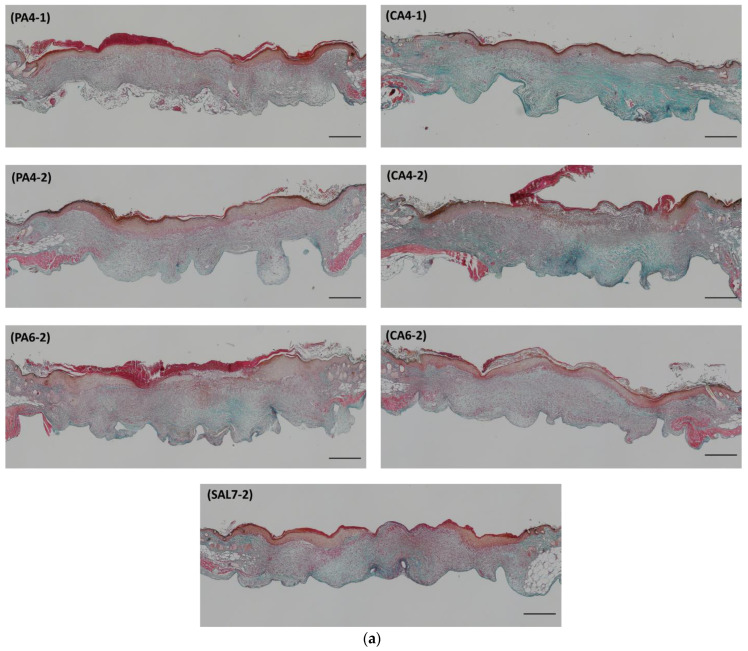

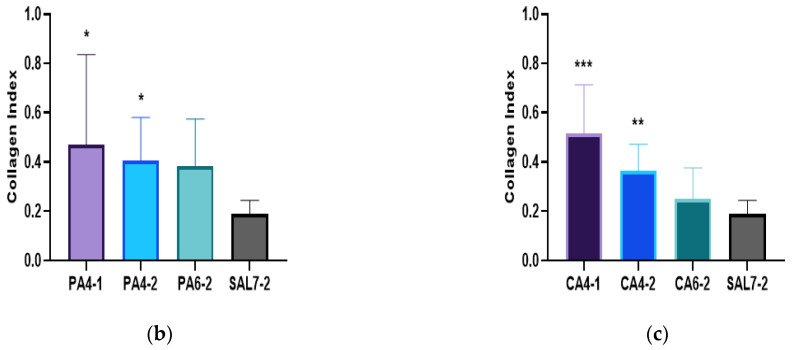
Evaluation of collagen deposition between phosphoric acid (PA) and citric acid (CA) treatment groups on day 7 post-wounding utilizing Masson Trichrome (MT) staining technique. (**a**) Histology analysis of skin sample obtained from all treatment groups. Original magnification 4×. Black scale bar represents 500 µm. (**b**) Collagen content evaluation between PA4-1, PA4-2, PA6-2 in comparison to SAL7-2 control group. (**c**) Differences in collagen index between CA4-1, CA4-2, CA6-2 and SAL7-2 control group. Each bar represents mean ± standard deviation. * *p* < 0.05, ** *p* < 0.01 and *** *p* < 0.001 indicates the results obtained were statistically significant from control group (SAL7-2).

Collagen fiber deposition and collagen density of collected wounded tissues were evaluated with Masson Trichrome (MT) staining technique, as shown in Figure 5. Wound tissues collected at day 7 showed a significantly higher density of collagen fibers when treated with phosphoric acid (PA) pH 4 and citric acid (CA) pH 4 once-daily treatment, showing collagen index of 0.47 ± 0.37 (PA4-1, *p*-value = 0.0103) and 0.52 ± 0.19 (CA4-1, *p*-value < 0.0001), followed by pH 4 once-every-second-day treatment with collagen index measured at 0.41 ± 0.17 (PA4-2, *p*-value = 0.0486) and 0.37 ± 0.11 (CA4-2, *p*-value = 0.0050) in comparison to control saline group (SAL7-2) measuring at 0.19 ± 0.05.

## 3. Discussion

Wound acidification using acidic buffers was demonstrated to be an effective treatment to increase re-epithelialization, wound closure, and collagen levels of non-infected wounds, all of which are indicative of improved wound healing. A previous study by our group established that the acidification regime once-every-second-day significantly improved metabolic activity and migration of keratinocyte and fibroblast skin cells. In vitro models were studied with acidic buffers at a pH range between 3 and 7 and both skin cells were observed to be more viable and to migrate faster in acidic buffers at pH 4, 5, followed by 6, with correlation to buffer ionic strength [24]. In this study, we found wounds treated with acidic pH 4 buffers following a once-every-second-day treatment with phosphoric and citric buffers showed complete wound re-epithelialization by day 7 of the treatment period, compared to saline solution. 

A faster wound re-epithelialization rate was observed in wounds treated by pH 4 buffers in both phosphoric and citric treatment groups compared to pH 6 buffers. This was evidenced from our histological analysis of epithelial thickness and wound closure measurements, showing wounds treated by pH 4 acidic buffers to have attenuated, thinner and more resolved epithelium, indicating better wound recovery. The transition of a wound epithelium thickness is evident of wound progression to the final maturation phase, due to halting migration of keratinocytes and the regeneration of a stratified epithelium, leading to a thinner epithelium [37] These wounds also showed narrower panniculus gap measurements coupled with shorter wound length by end of the treatment period, suggesting faster wound closure, potentially due to contraction by the panniculus carnosus muscle [38] compared to pH 6 treatments. Significantly shorter wound length measurements also depicted faster re-epithelialization in both acidic buffer treatment groups. We propose this contributed to the buffering capacity of the buffer systems that helps to maintain an acidic wound pH milieu to stimulate many cellular processes involving wound re-epithelialization which are enhanced by acidic pH [4,5,6,7]. The buffering capacity of a buffer system is dependent on both the acid pKa and pH value of the buffering system that allows the buffer to resist pH changes by an endogenous mechanism [39]. Therefore, this highlights the importance of acid choice in formulating a buffering system to be effective in resisting pH changes by endogenous processes to maintain an acidic wound milieu.

Overall, citric acid treatment groups (CA) were found to enhance wound healing, showing a better percentage of re-epithelialization, thinner epithelial layer, better wound contracture, and higher levels of collagen deposition in comparison to phosphoric acid (PA). This finding of slower healing by phosphoric acid treatment groups indicates that the acidic environment created by an acid with higher buffering strength slows down the healing activity of various intricate cellular processes, affecting rate of wound recovery. Interestingly, once-daily treatment by phosphoric acid (PA4-1) was observed to promote faster external epidermal wound recovery but slower internal wound contraction, evidenced from both macroscopic and histological evaluation. This is supported by the PA4-1 treatment group showing a higher percentage of re-epithelialization but larger panniculus gap measurement than other treatment groups indicative of slower wound contraction. This behavior is correlated to keratinocytes being able to tolerate more acidic conditions, which can be described by their role in the stratum corneum [7]. As the stratum corneum is constructed predominantly of keratinocytes, many studies have demonstrated that the acidic microenvironment of the skin milieu plays a key role in keratinocyte differentiation, especially during wound repair. Keratinocytes differentiation triggers an upregulation of ceramides synthesis, lipids, and aids in the restoration of lipid lamellae structure which are required to restore the epidermal layer [40,41,42]. Thus, this helps to explain the observed faster re-epithelization of the epidermal layer by once-daily phosphoric acid treatment at pH 4 (PA4-1), with contrasting slower internal tissue recovery. Furthermore, we also observed that wound contracture was less significant in phosphoric acid-treated groups compared to citric acid-treated wounds. This was evidenced from all citric acid groups showing significant thinner panniculus gap measurements than phosphoric acid-treated wounds, with only PA4-2 showing significant difference to the control group. We theorize faster re-epithelization can be achieved with the use of acids with stronger buffering capacity. However, cellular processes do not favor strong acid buffers and can impede wound tissue recovery rate.

The upregulation of the transforming growth factor-β (TGF-β) signaling pathway was considered as a potential mechanism by which phosphate from phosphoric acid and citrate from citric acid treatments stimulate the expression of these growth factors. Canonical activation of TGF-β occurs in the early stages of healing [43]. Upon activation, TGF-β play a crucial role in regulating fibroblast migration, proliferation and differentiation by binding to the heteromeric receptors complex transforming growth factor-β type I and type II receptors (TGFBR1 and TGFBR2) [44,45]. Phosphorylation of activin-like kinase 5 (ALK5), which is a type of TGFBR1, enables the induction of SMAD2 and SMAD3 receptor pathways that promotes differentiation of fibroblasts, resulting in increased production of myofibroblasts [46,47,48]. Wound closure is known to be facilitated by generated traction and contractile forces coordinated by surrounding myofibroblasts cells. This is an important step for remodeling of connective tissue to promote faster wound healing [26,49]. Our findings showed wounds treated with acidic buffers induced a faster rate of wound closure compared to the saline group. Mechanical stress by the extracellular matrix (ECM) is induced by migrating myofibroblasts and fibroblasts to the wounded site, which is also stimulated by acidification of the microenvironment that causes small tractional forces in newly formed granulation tissues [50]. Such increase in wound closure also explains our findings of high collagen levels within treated wound tissues, contributed by higher collagen synthesis by myofibroblasts cells [51,52].

Histological analysis of collected wound tissue treated by acidic buffers revealed high level of collagen deposition at the wounded site. Collagen is known to be a key component in the formation of an extracellular matrix contributing to mechanical strength and elasticity of repaired tissues [53,54]. As collected tissues were shown to be fully re-epithelialize by day 7, higher levels of collagen deposition at a later stage of the wound healing process is also evidence of increased wound closure and re-modelling activity when treated with acidic buffers, as previously described. Other studies have reported a similar increase in collagen levels at the wounded site when the wound pH microenvironment was modified using ascorbic acid treatments in murine models [55,56]. Therefore, this finding of higher collagen levels in treated tissues correlates to the increased rate of re-epithelialization and wound closure, as observed, suggesting that changing the wound environment to be more acidic leads to better wound healing.

## 4. Materials and Methods

### 4.1. Chemicals

Phosphoric acid, citric acid and sodium hydroxide were purchased from Sigma-Aldrich (Castle Hill, NSW, Australia). Sterilized saline solution was purchased from Ebos Healthcare (Kingsgrove, NSW, Australia).

### 4.2. Topical Buffer Preparation

The 0.01 M phosphoric acid solution (H_3_PO_4_) was prepared by gently pipetting 0.068 mL 85% *w*/*w* phosphoric acid to 25 mL sterile Milli-Q water. The solution was mixed thoroughly for 3 min via inversion mixing to ensure complete dissolution of the chemical, and the final volume was adjusted to 100 mL with sterile Milli-Q water. The 0.01 M citric acid solution (C_6_H_8_O_7_) was prepared by adding 0.19 g crystalline citric acid to 100 mL sterile Milli-Q water. The 0.01 M sodium hydroxide solution (NaOH) was prepared by adding 0.4 g crystalline sodium hydroxide to 1 L sterile Milli-Q water followed by inversion mixing as described above. 

Both phosphoric acid and citric acid buffers were prepared by adjusting pH values of 0.01 M phosphoric acid and 0.01 M citric acid with 0.01 M sodium hydroxide solution to pH values of 4 and 6, respectively. All acidic buffer solutions were incubated for 24 h and the pH values of all prepared buffers were confirmed with Orion Star A321 portable pH meter (ThermoFisher Scientific, Scoresby, VIC, Australia). All topical buffer solutions were autoclaved prior to surgery.

### 4.3. Murine Wound Repair Model

The murine study was carried out in compliance with current guidelines for the care of laboratory animals and the animal ethics application was approved by the University of South Australia Animal Ethics Committee (approval number U20-17). A total of 56 adult female BALB/c mice, with average age of 10 to 15 weeks and size of 19 to 25 g, were sourced from the Animal Resources Centre (ARC), and housed and acclimatized at the Core Animal Facility (CAF) for a minimum of 7 days. Animal feed was standard rat and mouse chow from Specialty Feeds. Mice were group-housed with *n* = 5 and maintained in standard laboratory conditions (temperature: 25 ± 2 °C, humidity: 55 ± 5%, 12 h/12 h light/dark cycle) prior to surgery and individually housed following wounding. 

Buprenorphine was given as an analgesic 30 min pre-operation and, following anesthetic induction using isoflurane gas to achieve surgical depth anesthesia, hair from the dorsal mouse skin was shaved with a sterile electric clipper and further depilated by applying hair removal cream for 1 min. To minimize infection, the mice’s skin was washed with sterile water and cleaned using 70% alcohol swabs. Two circular full-thickness skin wounds were induced using a sterilized AccuPunch 6 mm^2^ punch biopsy tool. With wound size not being considered as part of randomization, a simple randomization strategy with allocation concealment was utilized prior to surgery, with mice being simultaneously randomized into seven groups according to treatment regimens (*n* = 8). Group 1—phosphoric acid pH 4 buffer solution, one treatment daily (PA4-1), Group 2—phosphoric acid pH 4 buffer solution, one treatment every second day (PA4-2), Group 3—phosphoric acid pH 6 buffer solution, one treatment every second day (PA6-2), Group 4—citric acid pH 4 buffer solution, one treatment daily (CA4-1), Group 5—citric acid pH 4 buffer solution, one treatment every second day (CA4-2), Group 6—citric acid pH 6 buffer solution, one treatment every second day (CA6-2), and Group 7—sterilized saline solution pH 7, one treatment every second day (SAL7-2). The open wounds were left open without sutures and dressed with 1 cm × 1 cm gauze dressing treated with 1 mL buffered solutions for at least 5 min. A further 3 cm × 3 cm Tegaderm dressing (3M, Australia) was applied on top to secure the treatment below. The dressings were placed on mice for the duration of the whole study and were changed daily, or once-every-second-day, according to treatment regimens, whilst mice were placed under mild anesthetic with isoflurane gas for a period no longer than 5 min during dressing change. Mice were observed and checked daily through the duration of the experiment (7 days) for signs of distress and pain, including weight, appearance (dull/ruffled coat, signs of dehydration and hunched posture), behavior (abnormal behavior and reluctance to move), appearance of wounds (infection, inflammation and swelling) and loss of dressings. Skin tissue, including the wounds, were collected from 56 mice following humane killing by carbon dioxide asphyxiation and death, confirmed by cervical dislocation on day 7.

### 4.4. Macroscopic Wound Size Measurement

Macroscopic wound measurement was carried out on digital photographs of the wounds taken on each day during dressing change at day 0 (initial wound) and following measurements taken on days 2, 4, 6 and 7 (actual wound) using a ruler. Actual wound area was traced and normalized by calibrating each image using Image Pro Plus Software (Maryland, USA). The percentage of wound healing (% of initial) was calculated as below:

$$\frac{Area\;of\;initial\;wound-area\;of\;actual\;wound}{Area\;of\;initial\;wound}\;\times \;100\%$$

### 4.5. Histological Assessment by Hematoxylin & Eosin (H&E)

Skin tissue, including the wound and its surrounding skin tissues, were collected from mice following humane killing on day 7. Skin tissue was fixed for 24 h in 10% neutral formalin at room temperature, processed in Leica TP 1020 tissue processor (Leica Microsystems, North Ryde, NSW, Australia) and embedded in paraffin blocks. Skin tissue was sectioned at 5 μm using a microtome (Leica Microsystems, North Ryde, NSW, Australia) for histological assessment. 

Paraffin sections were dewaxed by immersion in xylene, followed by ethyl alcohol and tap water. Lillie-Mayer’s hematoxylin was then used to stain the sections for 6 min prior to rinsing with tap water until the water was colorless. Tissue sections were stained “blue” in 5% bicarbonate solution for 15 s prior rinsing the sections with tap water. Hematoxylin stain was differentiated in 0.25% acid alcohol before second incubation in 5% bicarbonate solution for 15 s. The sections were then stained using alcoholic eosin for 2 min, washed with ethanol prior to incubation with xylene and mounting in DPX mounting media (Sigma-Aldrich, Castle Hill, NSW, Australia).

The percentage re-epithelialization (%) was calculated based on total wound length (the area between the first hair follicle either side of the wound and above the break in the panniculus) at day 7 using the following formula:

$$\;\frac{Re-epithelialized\;wound\;length_{Day7}}{Total\;epithelialized+un-epithelialized\;wound\;length_{Day7}}\times 100\%$$

### 4.6. Histological Assessment by Masson Trichrome (MT)

The properties of connective tissues at the wound site, such as collagen, muscles and keratin, were evaluated using Masson Trichrome staining. Paraffin-embedded sections were firstly dewaxed by immersion in xylene and ethanol prior to rinsing with tap water. The sections were then placed in Bouin’s Fixative solution at 60 °C for 30 min before cleaning with water. The sections were placed in celestine blue followed by Lillie-Mayer’s hematoxylin for 3 min each before rinsing in running water until colorless. Prior to incubating the sections in Fuschin Ponceau for 5 min, the sections were briefly dipped in 5% bicarbonate solution for 15 s followed by washing in water. The sections were then incubated in 5% phosphotungstic acid and light green staining solution for 10 min and 3 min, respectively. All samples were briefly dipped in 1% acetic acid followed by washing in water. Dehydration of the sections was performed by briefly dipping the sections in ethanol and xylene before mounting with DPX mounting media (Sigma-Aldrich, Castle Hill, NSW, Australia).

For quantitative morphometric analysis, the slides were captured as RGB images and analyzed using Image J software (Version 1.32j, National Institutes of Health, USA). The number of blue/green pixels indicating collagen was then evaluated from the RGB images with a macro written by Kennedy et al., 2006 [57] by converting pixels of the image with substantially greater (>120%) blue than red intensity to have the new, grey scale amplitude = 1, leaving other pixels as with amplitude = 0.

### 4.7. Statistical Analysis

Statistical analysis was performed using GraphPad Prism v 8.0 (GraphPad Software, Inc., San Diego, CA, USA). Continuous variables were presented as a mean ± standard deviation and categorical variables as percentages. For wound healing (% initial), percentage re-epithelialization, wound length, epithelial thickness, panniculus gap measurement and collagen index, statistically significant difference were determined by one-way analysis of variance (ANOVA) with Dunnett’s multiple comparisons post-hoc test. For absolute wound size over time, two-way analysis of variance (ANOVA) coupled with Dunnett’s multiple comparisons post-hoc test was performed. Shapiro–Wilks was selected as the normality test for all studies. The level of statistical significance was set to *p* < 0.05.

## 5. Conclusions

This study has established the in vivo efficacy of acidification in promoting wound healing using acidic buffers at pH 4, following a once-every-second-day regimen in non-infected wounds. Wound acidification was demonstrated to promote a number of cellular responses, such as increasing the rate of re-epithelialization, wound closure, and collagen synthesis, inducing faster wound healing. Significant improvement to wound healing was observed as early as 2 days post-treatment. pH is shown to play a role in mediating the rate of wound healing as evidenced by faster wound healing by pH 4 buffers, compared to wounds treated by pH 6 buffers. Our study has shown the significance of acid choice, treatment frequency and duration; and the importance of maintaining an acidic wound microenvironment at pH 4 could therefore be a significant therapeutic strategy for improving wound management. 

## Data Availability

Data is contained within the article.
